# Deep learning-based optical approach for skin analysis of melanin and hemoglobin distribution

**DOI:** 10.1117/1.JBO.28.3.035001

**Published:** 2023-03-27

**Authors:** Geunho Jung, Semin Kim, Jongha Lee, Sangwook Yoo

**Affiliations:** Lulu-lab, AI R&D Center, Seoul, Republic of Korea

**Keywords:** skin analysis, deep learning, light–tissue interaction, melanin, hemoglobin, VISIA

## Abstract

**Significance:**

Melanin and hemoglobin have been measured as important diagnostic indicators of facial skin conditions for aesthetic and diagnostic purposes. Commercial clinical equipment provides reliable analysis results, but it has several drawbacks: exclusive to the acquisition system, expensive, and computationally intensive.

**Aim:**

We propose an approach to alleviate those drawbacks using a deep learning model trained to solve the forward problem of light–tissue interactions. The model is structurally extensible for various light sources and cameras and maintains the input image resolution for medical applications.

**Approach:**

A facial image is divided into multiple patches and decomposed into melanin, hemoglobin, shading, and specular maps. The outputs are reconstructed into a facial image by solving the forward problem over skin areas. As learning progresses, the difference between the reconstructed image and input image is reduced, resulting in the melanin and hemoglobin maps becoming closer to their distribution of the input image.

**Results:**

The proposed approach was evaluated on 30 subjects using the professional clinical system, VISIA VAESTRO. The correlation coefficients for melanin and hemoglobin were found to be 0.932 and 0.857, respectively. Additionally, this approach was applied to simulated images with varying amounts of melanin and hemoglobin.

**Conclusion:**

The proposed approach showed high correlation with the clinical system for analyzing melanin and hemoglobin distribution, indicating its potential for accurate diagnosis. Further calibration studies using clinical equipment can enhance its diagnostic ability. The structurally extensible model makes it a promising tool for various image acquisition conditions.

## 1 Introduction

The optical properties of human skin have been studied for various purposes. Skin layers are optically modeled in computer vision for a detailed description of the face.^1^^,^^2^ In dermatology, light absorption and scattering are the main principles for diagnosing skin conditions.^3^^,^^4^ In biomedical research, the distribution of chromophores and hemodynamic changes are imaged using diffuse optical tomography,^5^ optical coherence tomography,^6^ and spatial frequency domain imaging.^7^

In terms of skin analysis equipment, the optical measurement approach has various advantages compared to other imaging modalities: non-invasive, relatively safe, and real-time or frequent measurements. As examples of single-measurement devices, the Mexameter, Sebumeter, and Visiometer can be used to quantify pigmentation, oiliness, and wrinkles, respectively.^8^^,^^9^ Multifunctional systems utilize image processing techniques to skin images acquired from well-constructed optical systems with minimal ambient light. For example, VISIA, Robo skin analyzer, and DermaVision provide the ability to measure melanin, hemoglobin, wrinkles, and pores.^10^^–^^12^

Multifunctional systems analyze shape and color-related skin information based on the optical properties of human skin. The shape features, such as the skin surface and facial structure, are enhanced in the specular light images, and the wrinkles^13^^,^^14^ and pores^15^ belong to these features. Skin color is the result of the interactions between light and skin chromophores such as melanin and hemoglobin,^16^ and this feature is enhanced in cross-polarized images.^17^ For accurate measurements, clinical multi-functional systems are used to acquire and analyze skin images with ambient light blocked. Despite the fact that these professional systems have produced reliable analysis results and are widely used in clinics, they have several limitations: the analytical tool is dedicated to the image acquisition system. Images should be acquired in a specific environment and this result in a lack of versatility. The image acquisition system and analysis tools require a high cost, and the analysis process is time-consuming due to the complex calculations involved, such as fitting for each pixel. This is particularly important in medical applications that require high-resolution images, even if it needs high computational resources and time.

To alleviate those limitations, we propose a novel approach using a deep learning model trained to solve the forward problem of light–tissue interactions. The model is structurally extensible for various light sources and cameras and it maintains the resolution of the input image for medical applications. The proposed approach is evaluated with the professional clinical system, VISIA VAESTRO. In addition, this approach was applied to simulations with varying amounts of melanin and hemoglobin.

## 2 Methods

**Fig. 1:**
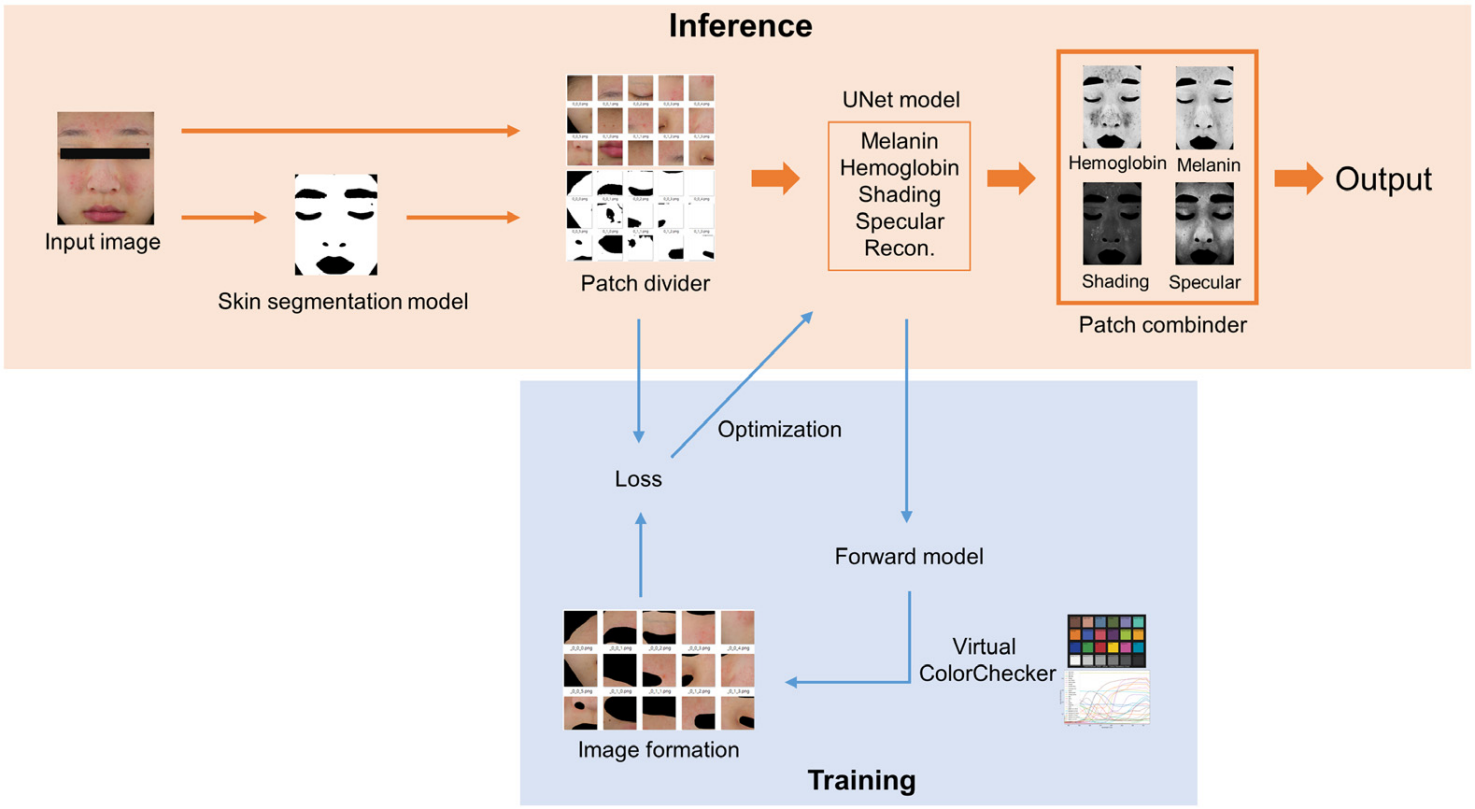
Diagram of the entire system: the training and inference processes.

The concept of the decomposition structure is based on the reference study in Ref. 18. The referenced structure has been modified and extended to include a skin segmentation model, patch divider and combiner, and virtual ColorChecker in Fig. 1 and verified for medical applications.

### 2.1 Skin Segmentation Model

As the forward model is based on the optical properties of the skin, it is necessary to separate non-skin areas, such as the background, eyes, nostrils, and lips. To extract the skin area from any face image, we developed a skin segmentation model using a lightened structure of the basic U-Net model with reduced number of channels. The model was trained using 380 images with an input and output size of 640×480  pixels, achieving a performance of CIoU 0.95.

### 2.2 Patch Divider and Combiner

When a high-resolution image is resized to the UNet input size, the detailed features of the image are lost. This loss could be a critical problem in medical applications where diagnostics require detailed characteristics. Therefore, the input image is divided into multiple patches to maintain the input image resolution. To remove borderlines that may appear after merging, the patches are combined by multiplying the overlapping areas by the gradient weight, and the overlapping area is added to the patch size. Since the image acquisition conditions for all patches are the same, the skin’s optical properties do not change even if analyzed in units of patches. In this study, the input image is divided into 4×6 patches with a size of 256×256  pixels for inference.

After dividing into patches, the gamma correction is inverted by Eq. 1 to make the image linear,

$$I_{\mathrm{rgb}}={\left(\frac{I_{\mathrm{srgb}}+0.055}{1.055}\right)}^{2.4},$$ (1)

where $I_{\mathrm{srgb}}$ is the input color image in the sRGB color space, while $I_{\mathrm{rgb}}$ is the corresponding image in the RGB color space with inverted gamma correction.

### 2.3 UNet Model and Training Conditions

**Fig. 2:**
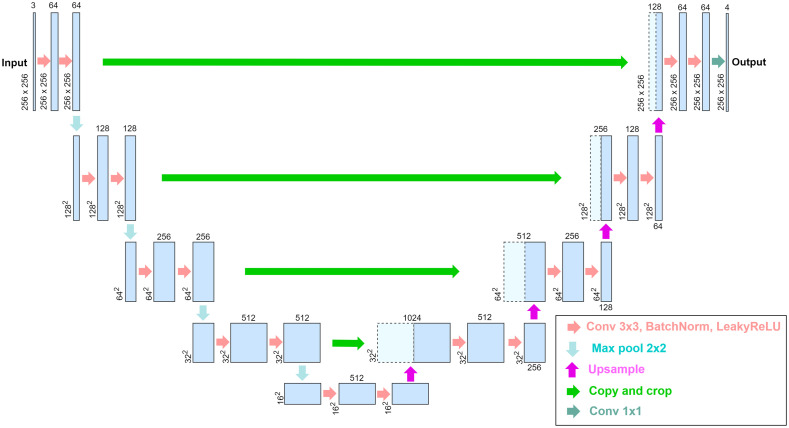
Architecture for the image decomposition: the UNet model.

Figure 2 shows the architecture of the UNet model used for image decomposition. For training, the model is initialized with the pretrained model for the Carvana dataset. The Adam optimizer is used with l2 weight decay and a cosine annealing scheduler. The mean squared error (MSE) is employed as a loss function, and the peak signal-to-noise ratio (PSNR) is used to estimate the similarity between reconstructed and original skin images. The python library “albumentation” is used to augment the training dataset by randomly flipping, scaling, and rotating it.

The proposed model decomposes a color image into four outputs: melanin, hemoglobin, shading, and specular maps, allowing for the separation of the face image into its color and morphological components. Shading is used to add depth to a flat image and create a three-dimensional appearance. Specular represents light that is directly reflected from the skin surface and includes morphological elements constituting the input image, such as surface shape and roughness, as well. This study primarily focuses on color-related skin components, melanin and hemoglobin maps. As skin color is also affected by morphological factors, shading and specular maps are incorporated into the structure to account for their effects.

#### 2.4 Forward Model

##### 2.4.1 Image acquisition

**Fig. 3:**
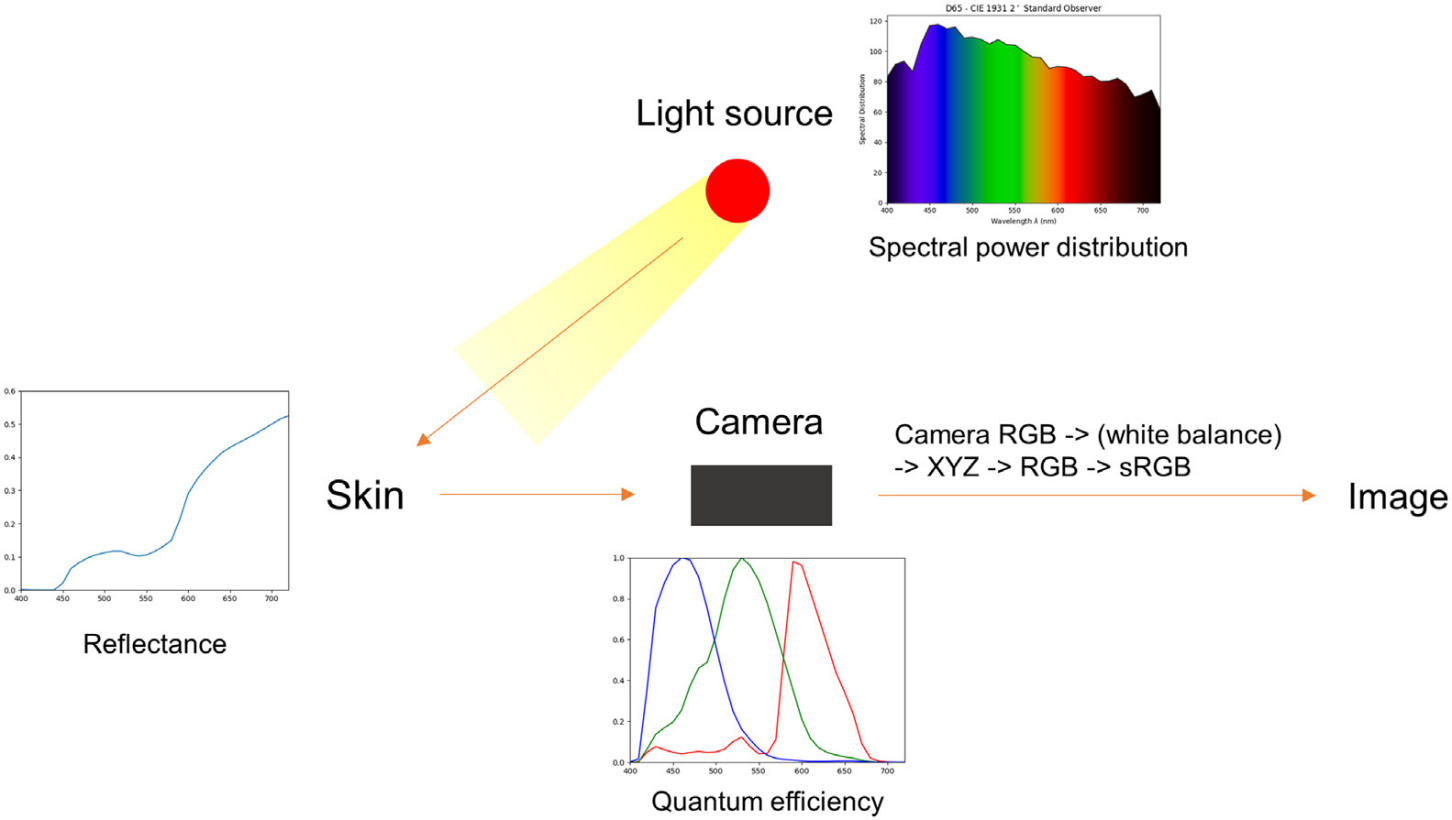
Schematic diagram of the skin image acquisition process.

Figure 3 shows a schematic diagram of the skin image acquisition process, in which light emitted from the source is reflected from the skin, detected by a camera, and formed into an image. The spectral characteristics of a light source, which cover all light that affects the image including ambient light, are expressed using spectral power distribution (SPD). In a typical measurement environment, the light source is a combination of various indoor lights and sunlight. In controlled conditions where ambient light is blocked, and images are acquired using professional equipment, the SPD of the light source of the equipment is supplied by the manufacturer’s specifications. In uncontrolled conditions, where images are acquired in an open environment, images are affected by various light sources, and the SPD can be measured directly using a spectrometer. Most of the light that illuminated the skin is diffused into the skin layers, and the rest is reflected from the surface. In the skin layers, visible light is mainly absorbed by melanin and hemoglobin and scattered exponentially as a function of wavelength.^19^^,^^20^ Skin reflectance is affected by various factors, including the scattering properties of the skin as well as the absorption of the melanin and hemoglobin, which have been extensively researched.^21^^–^^24^ The specular light and diffusely reflected light are detected as photons by the camera sensor and then converted into electrical signals according to the quantum efficiency of the camera to form an image. This image is in a RGB color space dependent on the camera device and is sequentially converted to the common color space XYZ, RGB, and sRGB.^25^

##### 2.4.2 Forward and inverse problem

Under a given light and skin reflectance, the diffusely reflected light is predicted as a unique solution to a forward problem. The radiative transfer equation (RTE), diffuse equation,^26^ or a stochastic methods Monte Carlo simulation^27^^,^^28^ can be used to solve this mathematical model. Acquiring skin images and analyzing skin components is the opposite process. It is difficult to obtain the actual values of skin properties because skin is a scattering-dominant material. Therefore, it has been predicted by solving the inverse problem using Inverse Monte Carlo,^29^^,^^30^ principal component analysis (PCA),^31^ independent component analysis (ICA),^32^ or polynomial fitting.^33^ During the fitting process, different skin property values are iteratively given, and the corresponding forward problems are solved until the error becomes lower than the threshold. It requires enough time to repeatedly calculate the forward problem for each pixel of the whole image. As the forward problem has been studied for a long time and applied to various studies,^34^^,^^35^ it is considered reliable and used to train the proposed model.

##### 2.4.3 Acquisition conditions

The image acquisition environment must be set in advance to proceed with the forward model. There are two factors: a light source and camera sensitivity. In this study, skin images were acquired using the “VISIA-CR” system. To ensure consistency between image acquisition and analysis conditions, the spectral data of the standard illuminant “D65” in ISO 2002 and the camera sensitivity of the “Canon 5D Mark II”^36^^,^^37^ within the wavelength range of 400 to 720 nm are used. The spectrum of the light source is normalized by inversely multiplying a scaling factor, which is derived by multiplying the light source and the maximum sensitivity value of the camera channels.

##### 2.4.4 Optical modeling of skin layers

The epidermis is the outermost layer of the skin. In the epidermis layer, light is absorbed by melanin and scattered by keratin fibers. However, the change in light direction could be ignored because of the thin thickness. In the dermis layer, light mainly experiences absorption in the hemoglobin of the capillaries and blood vessels, and scattering in collagen fibers and elastin fibers.^38^ Therefore, skin layers are optically modeled as follows: the epidermis is modeled as a melanin absorption layer, and the dermis is modeled as a combination of a hemoglobin absorption layer and multiple scattering layers. The measured light includes both the specular reflected light from the skin surface and the diffusely reflected light that is absorbed in the epidermis and dermis layers.

##### 2.4.5 Skin reflectance

The Kubelka–Munk theory is one of the popular methods for modeling light reflection in skin layers.^39^^,^^40^ Assuming that light is reflected only from the dermis layer, the simplified form of the theory is represented as follows:

$$R(\lambda )=T_{\text{epidermis}}(\lambda {)}^{2}R_{\text{dermis}}(\lambda ),$$ (2)

where $T_{\text{epidermis}}$ represents the fraction of light that transmitted the epidermis, and $R_{\text{dermis}}$ refers to the fraction of light reflected from the dermis layer.

The absorption and reduced scattering coefficient are shown in Eqs. (3) and (4).^41^ In a method which precisely measures in a wider wavelength range, such as diffuse reflectance spectroscopy, more chromophores can be included in Eq. (3), such as met-hemoglobin, water, lipid, and beta-carotene^42^^,^^43^

$$\mu_{a}(\lambda )=\Sigma_{i}S_{i}\epsilon_{i}(\lambda )C_{i},$$ (3)

$$\mu_{s}^{\prime }(\lambda )=a(f_{\text{Ray}}{\left(\frac{\lambda }{\lambda_{0}}\right)}^{-4}+(1-f_{\text{Ray}}){\left(\frac{\lambda }{\lambda_{0}}\right)}^{-b_{\text{Mie}}}),$$ (4)

where $S_{i}$ is the volume fraction, $\epsilon_{i}$ is the extinction coefficient, $C_{i}$ is the concentration for i in melanin and hemoglobin, a is the scattering amplitude, $b_{\text{Mie}}$ is the scattering power of Mie scattering, and $f_{\text{Ray}}$ is the fraction of scattering events as Rayleigh scattering.

The reflectance can be obtained through the diffusion equation.^42^ In this study, the skin reflectance calculated from Refs. 18, 34, and 44 is used based on the Kubelka-Munk theory. The volume fraction of the melanin is limited to a range of 1.3% to 43%, and the hemoglobin is in the range of 2% to 7%.^45^^–^^47^

##### 2.4.6 Image formation

The camera image measured by the sensor is described for each color channel m

$$I_{m}=\int_{\lambda_{\min}}^{\lambda_{\max}}L(\lambda )R(\lambda )C_{m}(\lambda )d\lambda ,$$ (5)

where L(λ) is the SPD of the light source, R(λ) is the skin reflectance, $C_{m}(\lambda )$ is the camera sensitivity of color channel m∈{r,g,b}. In this study, $\lambda_{\min}=450\;\mathrm{nm}$ and $\lambda_{\max}=750\;\mathrm{nm}$.

The light entering the camera is affected by the light source and skin reflectance [L(λ)R(λ) in Eq. (5)] and consists of specular and diffusely reflected light

$$I_{m}=\int_{\lambda_{\min}}^{\lambda_{\max}}(I_{d(m)}(\lambda )+I_{s(m)}(\lambda ))C_{m}d\lambda ,$$ (6)

$$I_{d(m)}(\lambda )=L(\lambda )R(\lambda )M_{\text{shading}}(\lambda ),$$ (7)

$$I_{s(m)}(\lambda )=L(\lambda )M_{\text{specular}}(\lambda ),$$ (8)

where $M_{\text{shading}}$ and $M_{\text{specular}}$ are the model outputs, the shading and specular maps; $I_{d(m)}(\lambda )$ and $I_{s(m)}(\lambda )$ are the diffuse and specular images for each color channel m; and $I_{\mathrm{cam}}=[I_{r},I_{g},I_{b}]$ is the camera image. The diffusely reflected light is influenced by the geometry of the face and the skin reflectance through optical interaction with the skin components in Eq. (7), whereas the specular light is directly reflected from the skin surface and is significantly affected by the geometry of the face in Eq. (8).

##### 2.4.7 Color transformation matrix with virtual ColorChecker

**Fig. 4:**
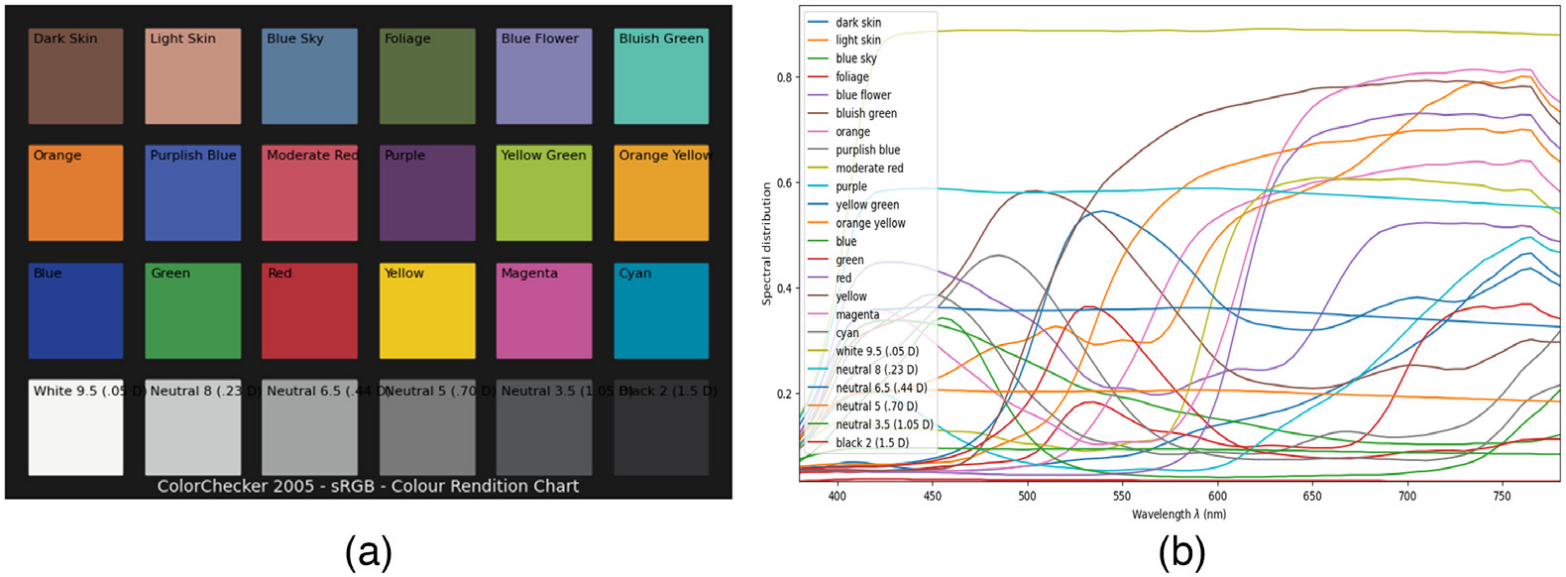
(a) Plate of the ColorChecker 2005 and (b) its reflectance measurements in Ref. 48.

A transformation matrix is required to convert between different color spaces. Considering its applicability in various acquisition systems and environments, the reflectance of the 24-patch version of ColorChecker^48^ is employed in Fig. 4. Given a light source and ColorChecker, it is possible to theoretically compute the RGB value of the camera and the XYZ (CIE 1931 2 degree standard observer) value for each color patch. The transformation matrix between different color spaces is derived by polynomial expansion.^49^ In this study, this method is specified as the “Virtual ColorChecker” and implemented using the Python Colour-Science library.

##### 2.4.8 Image formation

White balance is a normalization process that divides the sensor’s intensity by the value when the light source is directly detected $l_{m}$

$$[\begin{array}{c} I_{wb(r)}\\ I_{wb(g)}\\ I_{wb(b)} \end{array}]=[\begin{array}{ccc} 1/l_{r} & 0 & 0\\ 0 & 1/l_{g} & 0\\ 0 & 0 & 1/l_{b} \end{array}][\begin{array}{c} I_{r}\\ I_{g}\\ I_{b} \end{array}].$$ (9)

The color space of the camera image is an inherent characteristic of a camera device. It should be converted to the common color spaces XYZ, RGB, and sRGB, sequentially. The color space is converted by multiplying the color transformation matrix, and the sRGB image is obtained by applying gamma correction to RGB in Eq. (10). In this study, it is assumed that a minimally processed image captured by the camera is used

$$I_{\mathrm{srgb}}=1.055I_{\mathrm{rgb}}^{\left(1/2.4\right)}-0.055,$$ (10)

where $I_{\mathrm{rgb}}$ is an image in the RGB color space, and $I_{\mathrm{srgb}}$ is the gamma corrected image in the sRGB color space.

#### 2.5 Metrics

To quantify the performance of the proposed model, three methods are employed in this study: the MSE, PSNR, and the Pearson product-moment correlation coefficients.^50^^,^^51^ For training and validation, the MSE and PSNR are used to estimate the similarity between the input and reconstructed images, indirectly improving the melanin and hemoglobin analysis ability. After training, the correlation coefficient is used to directly compare the results with the clinical equipment, VISIA system. Since the VISIA system produces single-color distribution maps with red and brown for hemoglobin and melanin, respectively, they are converted to grayscale and employed for comparison.

#### 2.6 Experiments

The polarized image dataset was acquired from 198 subjects using the VISIA acquisition system, VISIA–CR. The dataset was divided into 2 groups: 168 subjects for model training and 30 subjects for testing the performance of the trained model. Facial images were collected with informed consent from all subjects, in accordance with all relevant national regulations and institutional policies, and approved by the authors’ institutional committees. The training images are divided into 20 patches (4×5) of 310×310  pixels and randomly cropped to 256×256  pixels during augmentation for reducing overfitting and improving robustness of the model. For testing, the images are divided into 24 patches (4×6) of 256×256  pixels. These patches are used as inputs to the model, and the output patches are combined to form a full image. To avoid the occurrence of blurry lines on the border of output patches, boundary areas are overlapped by 15 pixels and combined by multiplying the opposite gradient weights. Including the overlapping boundary areas, the combined output size is 979×1461  pixels. Finally, the proposed model and VISIA VAESTRO system are compared to analyze the correlation of the two system measurements for melanin and hemoglobin. The VISIA VAESTRO system is the integrated skin analysis toolkits including the melanin and hemoglobin analysis modules, RBX-Brown and RBX-Red Processing 1.0.^52^^,^^53^ To avoid repetition of software name throughout this manuscript, we will use the term VISIA system to refer to this analysis system. In addition, this approach is applied to simulated images with varying amounts of melanin and hemoglobin.

## 3 Results

**Fig. 5:**
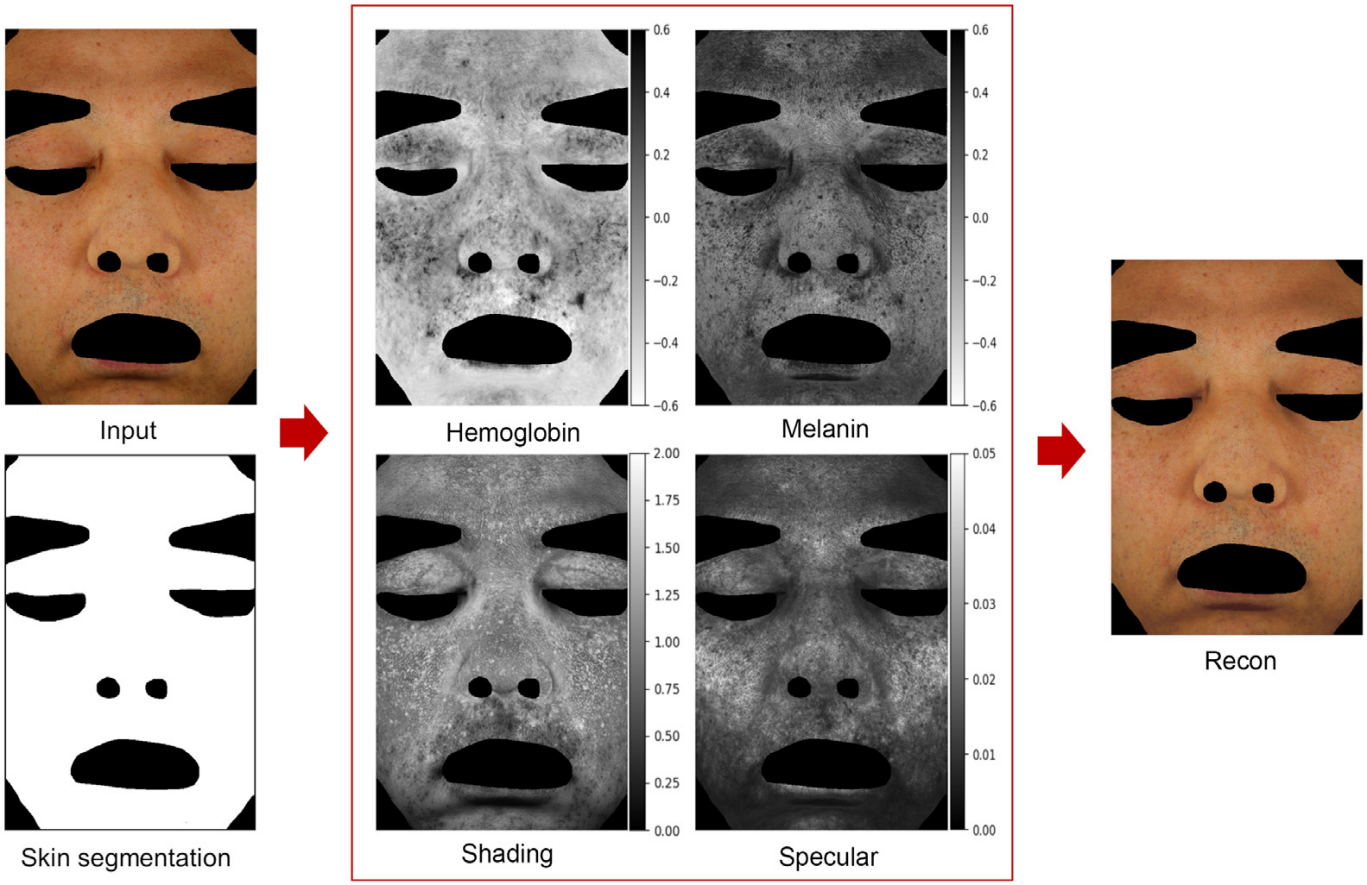
Decomposed (inverted in hemoglobin and melanin) and reconstructed images produced by the proposed model.

**Fig. 6:**
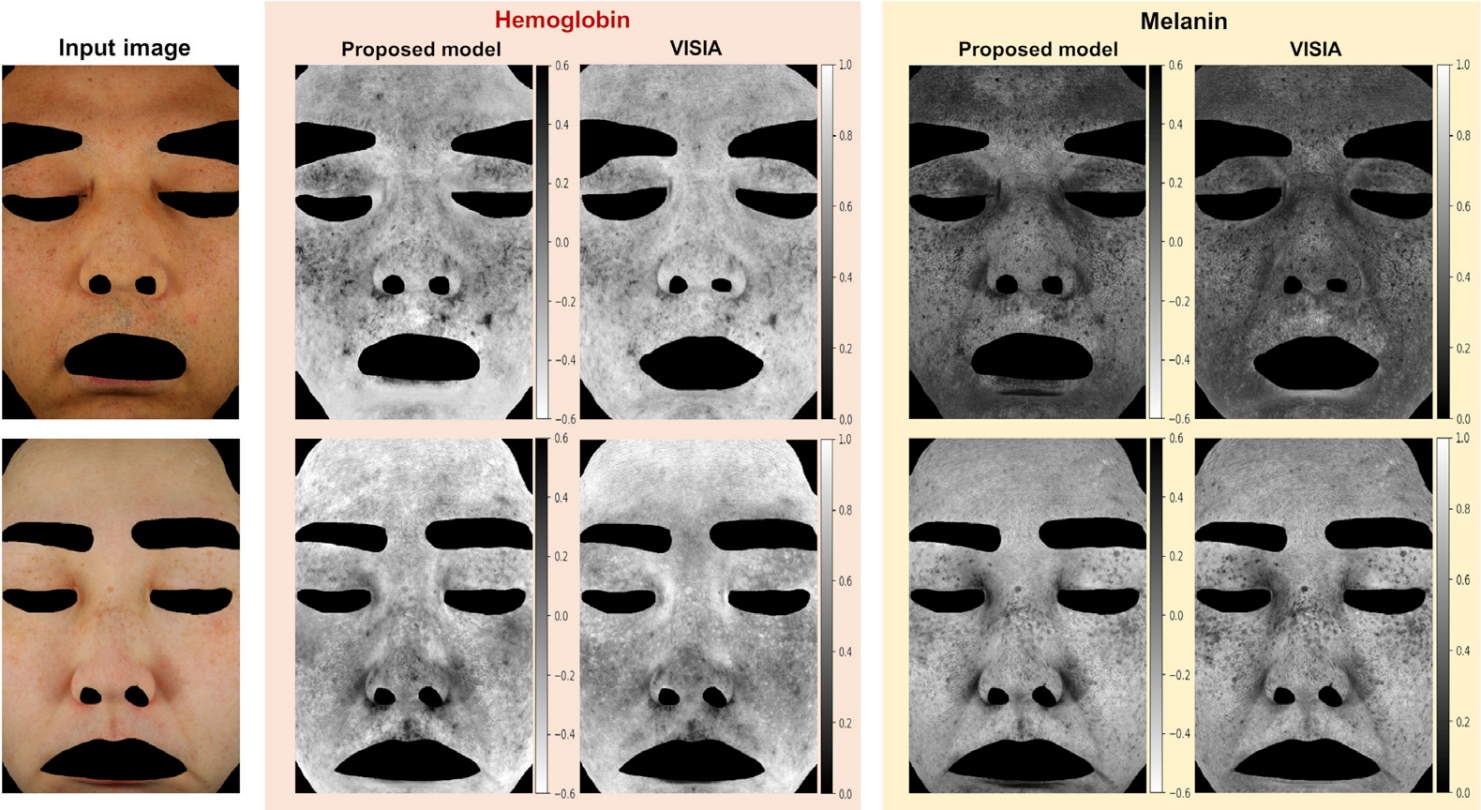
Hemoglobin and melanin analysis results from the proposed model (inverted) and VISIA system.

**Fig. 7:**
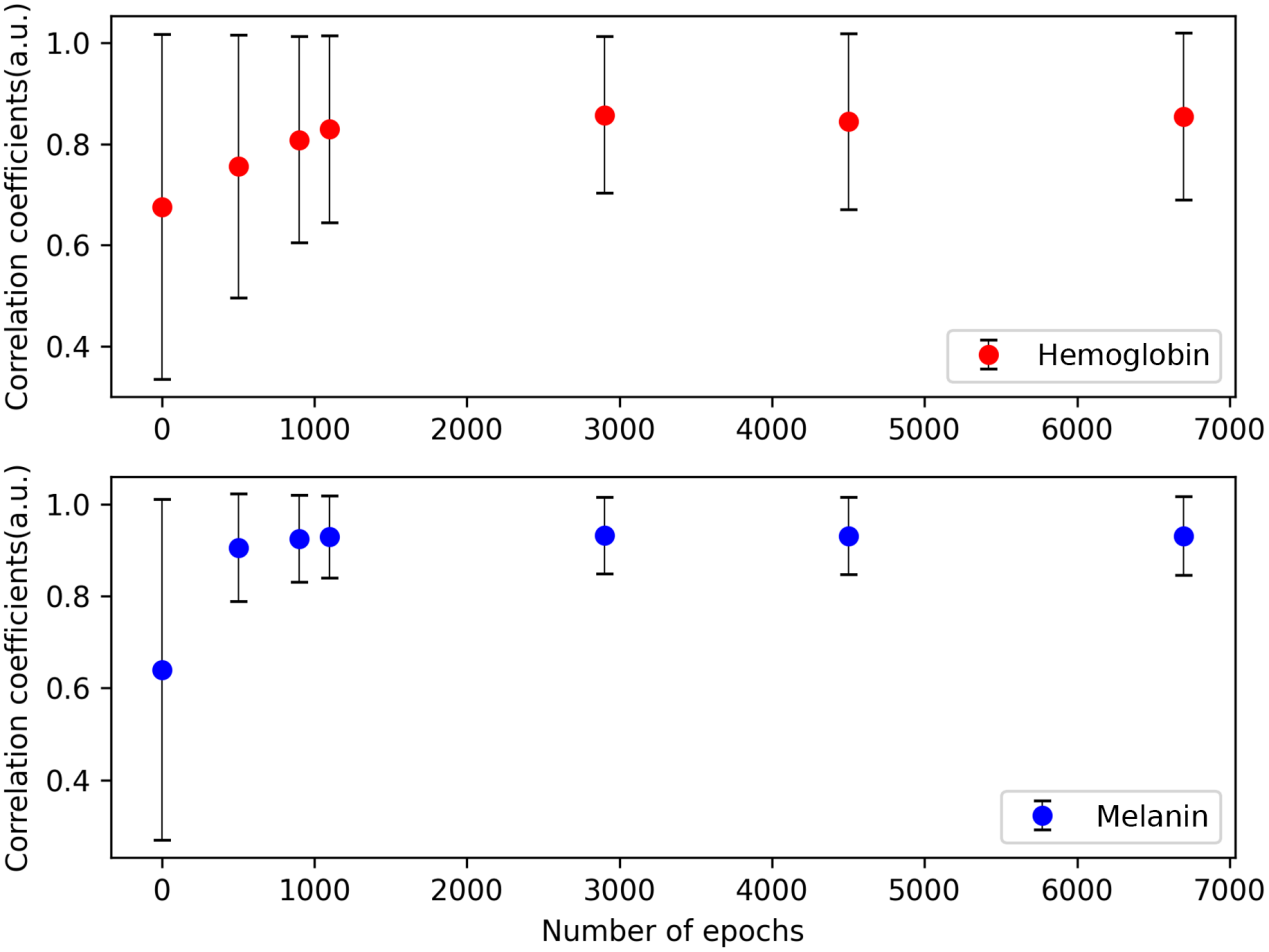
Correlation coefficients between the proposed model and the VISIA system depending on the number of epochs in training.

The proposed model decomposes the skin segmented area into hemoglobin, melanin, shading, and specular components that are combined to reconstruct the skin image, as shown in Fig. 5. The trained model achieved an average PSNR value of 42.7, and an example of the input and reconstructed images is shown in Fig. 5. The analyzed results of the hemoglobin and melanin obtained from the proposed model and the VISIA system are shown in Fig. 6 and their correlation coefficients according to training epochs are shown in Fig. 7. As the hemoglobin and melanin values increase, the intensity in grayscale image decreases in the VISIA system, while it increases in the proposed method. To facilitate comparison between the two methods, the grayscale images produced by the proposed method are inverted in Figs. 5 and 6. The results of the proposed model for seven different epochs are postprocessed and compared to the VISIA system. The correlation coefficient is 0.932 (std 0.083) for melanin and 0.857 (std 0.155) for hemoglobin in 2900 epochs.

**Fig. 8:**
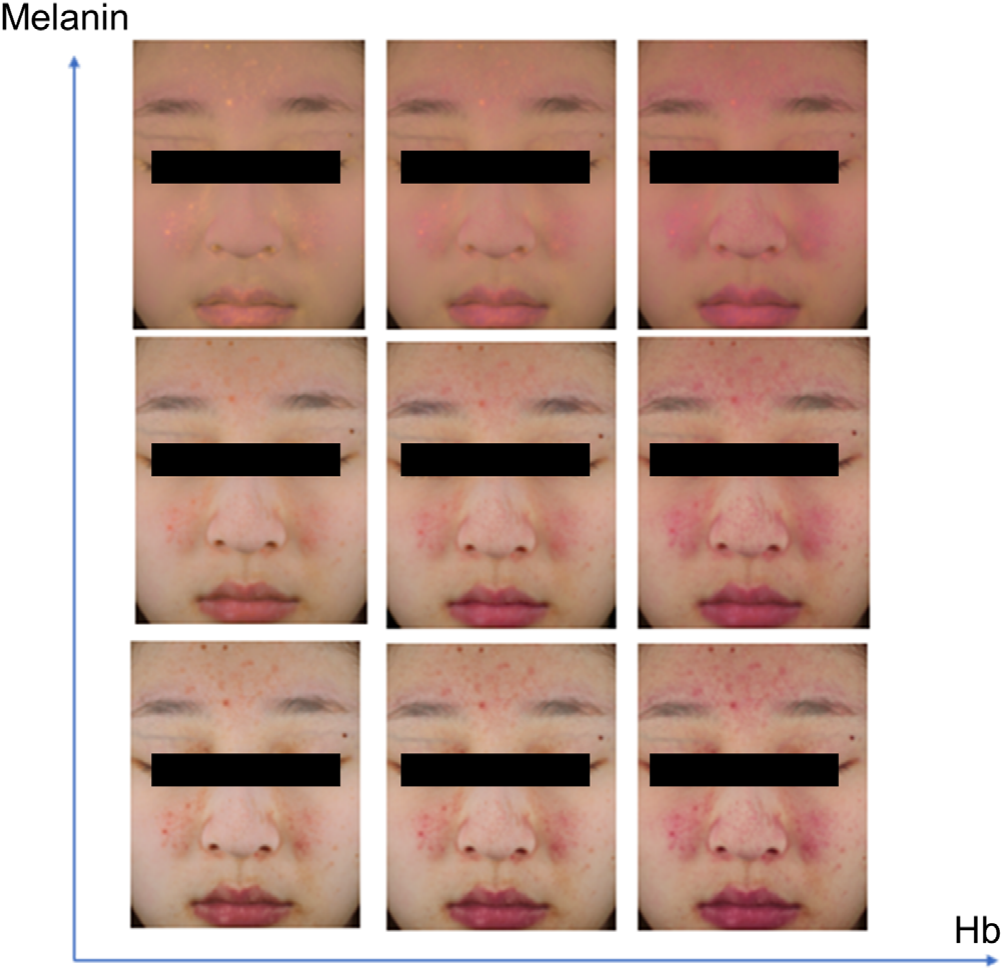
Simulated images for melanin and hemoglobin.

The simulated images by manipulating its melanin and hemoglobin levels in the skin are shown in Fig. 8. The simulated images are generated by modifying the center image with changes to melanin (multiplying by −1.2 and +1.2) and hemoglobin (adding −1 and +1) levels.

## 4 Discussion

The average PSNR value of the trained model exceeds 30, which indicates a high degree of similarity^54^ between the input and reconstructed images, shown in Fig. 5. The proposed model and the VISIA system show a similar tendency in Fig. 6, especially the melanin map has a higher overall value in (upper) dark skin than in (lower) lighter skin. The exact value of human skin properties is difficult to determine. Therefore, the proposed approach’s performance is evaluated by comparing it with one of the most reliable clinical equipment systems, the VISIA system.^11^^,^^52^^,^^55^ This implies that the correlation values are a comparison to determine the strength and direction of the relationship between the two systems, rather than an absolute accuracy of skin measurements. The proposed model is considered sufficiently trained at around 200 epochs, with correlation coefficients of 0.932 and 0.857 for melanin and hemoglobin, as shown in Fig. 7. Considering that image processing and deep learning are different approaches and the results of the VISIA system are not used in training, this strong positive relationship with clinical equipment can be interpreted as the light–tissue interactions being learned well as intended.

**Fig. 9:**
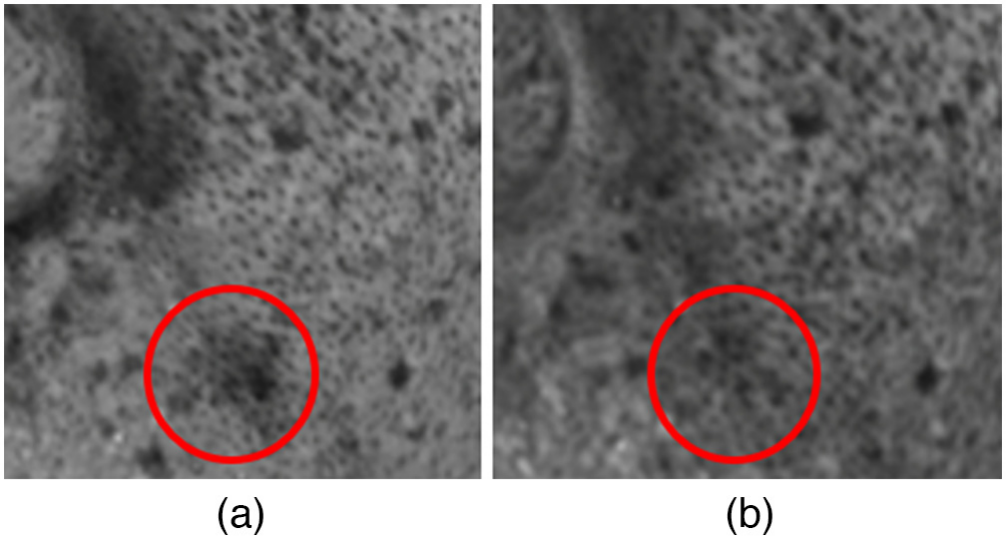
Magnified area of the melanin map from (a) the proposed model and (b) the VISIA system.

However, the VISIA system outperforms our proposed model, especially in terms of capturing detailed features. As an example, Fig. 9 shows the right corner of the mouth area from the melanin maps of the upper line in Fig. 6. When considering the input image, it is reasonable that the red spot in the result of the proposed method should not be emphasized like in the VISIA system. When the correlation coefficient is calculated by excluding the red spot, the difference in the coefficient is only 0.002. This insignificant value indicates that small differences in detailed areas are challenging to capture using overall representative values.

Besides the results, there are factors that can potentially affect the correlation values. The overall pixel values in the image can change depending on the plotting range when converting the analysis results into an image. This issue applied to both the proposed model and the VISIA system. To mitigate it, we conducted the analysis with constant plotting options for both systems. The VISIA system used the optimal conditions determined through postprocessing as a constant in the analysis process. To compare the analysis results of both systems, we calculated the correlation coefficients of the entire image rather than comparing the pixel intensity alone. This approach minimizes the effects caused by this issue. Next, the format of the result image differs between the single-color distribution map of the VISIA system and the grayscale image of the proposed model. It is possible that a slight difference may occur in the process of converting the color image to grayscale for the VISIA system.

The proposed approach has demonstrated the potential for generating simulated images with varying amounts of melanin and hemoglobin, as shown in Fig. 8. While changes in melanin and hemoglobin levels do affect skin tone, it is important to note that further research and verification are needed to accurately represent these changes in simulated images.

For diagnostic purposes, the following studies could be conducted. The forward model applied in the training part aims to find the absolute amount of skin components. If the range of output values and plotting options are calibrated with clinical equipment that supplies absolute values, such as Mexameter, the proposed model could be used for evaluating the severity of the disease. Furthermore, this study primarily focuses on color-related information, while the morphological characteristics are mainly utilized for accounting for color changes caused by shape-related features. If a follow-up study is conducted to enhance the morphological features of the specular map by modifying the learning process or applying postprocessing, the proposed method could be extended to the diagnosis of wrinkles and pores. Finally, the proposed model requires prior knowledge of the light source and camera information. If an additional algorithm is applied to estimate that spectrum information, the range of application will be expanded to images acquired from uncontrolled environments.

To enhance the performance of the proposed model, the following methods are considered. First, using the results of the VISIA system as ground truth (GT) during training can potentially improve the model’s performance by providing detailed information about skin properties. However, in this study, we intentionally chose not to use any GT for skin images to focus on investigating the performance of the proposed model in implementing light interaction with the skin. Using GTs is a standard practice for training deep learning models, and modifying the proposed model to include GTs will require extensive trials to optimize performance while preserving the advantages of both the current method and GTs. Second, in this study, a lightweight version of the basic U-Net model is employed to ensure efficient computational time. However, if the model prioritizes high performance over shorter analysis time and memory capacity, it may be worth trying model structures that use more parameters and take longer computational time but have superior performance. Finally, training with a large number of datasets and measuring accurate SPD using a spectrometer can help improve accuracy.

## 5 Conclusion

In this study, we trained a deep learning model to solve the forward problem of light–tissue interactions and its performance was evaluated using professional clinical equipment, the VISIA system. The model is structurally extensible for various acquisition conditions, and the skin segmentation model, patch divider and combiner, and virtual ColorChecker methods are applied for medical applications. The results showed a high correlation coefficients for melanin and hemoglobin. In addition, this approach was applied to simulated images with varying amounts of melanin and hemoglobin. It is expected that the proposed approach will be further developed for skin analysis and disease diagnosis through calibration studies using clinical equipment, ultimately providing a valuable tool for dermatologists and clinicians.

## Code, Data and Materials Statement

The code associated with this article is available upon request sent to the corresponding author. The image dataset is subject to institutional restrictions and can only be used for research purposes with the prior consent of the subjects and the approval of the author’s institutional committees. These restrictions are in place to protect the privacy and confidentiality of the subjects.
